# Angiogenesis in mammary Paget disease: histopathological analyses of blood vessel density and angiogenic factors

**DOI:** 10.1186/s13000-020-00988-y

**Published:** 2020-06-11

**Authors:** Yuri Akishima-Fukasawa, Naoko Honma, Hideaki Ogata, Yoshikiyo Akasaka, Tetuo Mikami

**Affiliations:** 1 Department of Pathology, Toho University School of Medicine, 5-21-16, Omori-Nishi, Ota-ku, Tokyo, 143-8540 Japan; 2 Division of Breast and Endocrine Surgery (Omori), Department of Surgery, Toho University School of Medicine, Ota-ku, Tokyo, 143-8540 Japan

**Keywords:** Mammary Paget disease, Angiogenesis, Blood flow, Basic fibroblast growth factor (bFGF), Vascular endothelial growth factor A (VEGFA), Immunohistochemistry

## Abstract

**Background:**

We examined the vascularity of mammary Paget disease histologically to confirm the increased blood flow observed previously by clinical imaging. The relationships among blood vessel density (BVD), histopathological parameters of blood flow in the nipple, and the expression of angiogenic factors such as basic fibroblast growth factor (bFGF) and vascular endothelial growth factor A (VEGFA) were examined.

**Methods:**

We calculated the average CD34-positive BVD and podoplanin (D2–40)-positive lymphatic vessel density (LVD) and the proportion of proliferating of endothelial cells in 14 Paget disease, 3 dermatitis biopsy, and 14 age-matched control cases. As a parameter related to blood flow in the nipple, the total CD34-positive blood vessel lumen area relative to the entire nipple area was measured in each Paget disease and control case using an automated image analysis system. Immunohistochemical expression of bFGF and VEGFA in Paget cells was also examined.

**Results:**

The average BVD and LVD were significantly higher in the Paget disease cases than in the dermatitis (*p* = 0.003) and control (*p* < 0.001) cases. The proportion of proliferating endothelial cells was also increased in the Paget disease cases. The ratio of the CD34-positive blood vessel lumen area to nipple area was also significantly higher in the Paget disease than control cases (*p* = 0.003). The average BVD was correlated with the average LVD (*r* = 0.734, *p* < 0.001) and ratio of the blood vessel lumen area to nipple area (*r* = 0.692, *p* < 0.001). Immunohistochemical expression of bFGF was strong in all Paget disease cases, with a significantly higher expression score in the Paget disease than dermatitis (*p* = 0.003) and control (*p* < 0.001) cases. The bFGF, but not VEGFA, expression score, was strongly correlated with the average BVD (*r* = 0.818, *p* < 0.001) and ratio of the blood vessel lumen area to nipple area (*r* = 0.503, *p* = 0.006).

**Conclusion:**

These results provide direct histopathological evidence of a marked increase in nipple blood flow in Paget disease detected by clinical imaging. bFGF is considered to play a pivotal role in angiogenesis in mammary Paget disease.

## Background

Paget disease was first described by James Paget in 1874 in a patient with breast cancer [1, 2]. A number of mammographic abnormalities in the breast may be found in Paget disease [3]. However, mammography is not always a reliable procedure for detecting Paget disease. Contrast-enhanced magnetic resonance imaging (MRI) is useful for detecting Paget lesions, because numerous vessels in the nipple/areola region provide a high contrast effect [4, 5]. Recently, our group used Doppler sonography to reveal proliferation of blood vessels in the nipple in Paget disease, and found that the blood flow and capillary density were significantly higher in Paget disease lesions than in dermatitis [6]. However, the histological research on Paget disease vascularity is insufficient [6, 7].

Several angiogenic and anti-angiogenic factors, including basic fibroblast growth factor (bFGF) and vascular endothelial growth factor A (VEGFA), regulate tumor angiogenesis. bFGF and VEGFA have been identified as key mediators of tumor angiogenesis, and overexpression of bFGF and VEGF has been found in a variety of human cancers. Xu et al. showed that bFGF and VEGF expression, detected by immunohistochemistry and quantitative reverse-transcription polymerase chain reaction, was significantly higher in extramammary Paget disease tissues than in adjacent normal tissues [2]. Chen et al. also showed cytoplasmic staining of VEGF in extramammary Paget disease [8], whereas, Ellis et al. reported that VEGFA was not expressed in Paget disease [9]. The immunohistochemical expression of VEGFA is controversial, and those studies did not investigate the relationships between these growth factors and vascularity [2, 8, 9].

Increased blood flow in the nipple on MRI and Doppler sonography has become a diagnostic marker for mammary Paget disease [4–6]; however, detailed histopathological confirmation is needed. We investigated the number of CD34-positive blood vessels and podoplanin (recognized by the D2–40 antibody)-positive lymphatic vessels in nipple tissue in Paget disease, dermatitis, and healthy tissues. We also examined the total area of CD34-positive vessel structures to estimate the blood flow volume in Paget disease and normal nipple tissues. Subsequently, we analyzed the relationships between these vascular parameters and angiogenic factors such as bFGF and VEGFA using immunohistochemistry to determine the mechanism of angiogenesis in mammary Paget disease.

## Methods

### Tissue specimens

We searched the pathology files of Toho University Omori Medical Center during 2004–2015 and identified 14 cases of mammary Paget disease, including 7 cases of Paget disease without ductal carcinoma in situ or invasive ductal carcinoma (pure Paget disease), 2 cases of Paget disease with ductal carcinoma in situ, and 5 cases of Paget disease with invasive ductal carcinoma. As a control group, normal nipple tissue was obtained from the surgical materials of 14 breast cancer cases. In addition, three biopsy specimens from nipple or areola dermatitis cases were also collected during the same period (2004–2015); these biopsies had been performed to rule out mammary Paget disease, yielding a diagnosis of dermatitis. All patients were women. The 14 control cases were selected according to consecutive case records and age matched to the Paget disease cases. All patients were diagnosed according to clinical and histopathological findings. This study was approved by the Ethics Committee of Toho University School of Medicine (No. 27130). Breast tissues were fixed in 10% buffered formalin, embedded in paraffin, cut into 3-μm-thick sections, and stained with hematoxylin and eosin for histopathological examination.

### Immunohistochemistry

The sections cut from the paraffin-embedded tissues including the nipple and nearby skin were used for immunostaining. After deparaffinization, the sections were pretreated, incubated with monoclonal antibodies (Table 1), and processed using the EnVison+ kit (Dako, Glostrup, Denmark) in accordance with the manufacturer’s instructions. Staining was visualized using diaminobenzidine tetrahydrochloride, and the sections were counterstained with hematoxylin. For CD34 and Ki-67 double staining, the sections were incubated with an anti-Ki-67 antibody (Table 1), processed using the EnVison+ kit, and stained with DAB. After rinsing in Tris-buffered saline, the sections were incubated for 1 h in 0.1 mM citrate buffer (pH 6.0) at 95 °C using a hot pot. Then, the sections were incubated with an antibody against anti-CD34 (Table 1), incubated with Histofine Simple Stain AP (M) (Nichirei, Tokyo, Japan) at room temperature for 30 min, visualized using the New Fuchsin Substrate kit (Nichirei), and counterstained with hematoxylin.

**Table 1:** Antibodies used in this study

Antibody	Clone	Host	Source	Dilution	Incubation	Pretreatment
Anti-CD34	QBEnd-10	Mouse	Dako	1 / 100	Overnight / 4 °C or 60 min / RT (double staining)	Microwave (CB)
Anti-podoplanin	D2–40	Mouse	Dako	1 / 50	30 min / RT	No pretreatment
Anti-αSMA	1A4	Mouse	Dako	1 / 100	30 min / RT	No pretreatment
Anti-cytokeratin 7	OV-TL12/30	Mouse	Dako	1 / 100	Overnight / 4 °C	Electric pot (95 °C, TRS)
Anti-Ki-67	MIB-1	Mouse	Dako	1 / 100	60 min / RT	Pressure cooker (TRS)
Anti-bFGF	20B242.2	Mouse	PeproTech	1 / 400	60 min / RT	Electric pot (95 °C, TRS)
Anti-VEGFA	SP28	Rabbit	Abnova	Ready to use	60 min / RT	Electric pot (95 °C, CB)

Abnova (Taipei, Taiwan); Dako (Glostrup, Demmark); PeproTech (Rochy Hill, NJ)

*bFGF* Basic fibroblast growth factor, *CB* Citrate buffer, *RT* Room temperature, *SMA* Smooth muscle actin, *TRS* Target Retrieval Solution (Dako, pH 9), *VEGFA* Vascular endothelial growth factor-A

### Vessel density and evaluation of endothelial cell proliferation

CD34-positive vessel structures were judged as blood vessels, and podoplanin (D2–40)-positive vessel structures were judged as lymphatic vessels. The average blood vessel density (BVD) and lymphatic vessel density (LVD) were measured at the nipple surface just beneath the basement membrane of the epidermis to approximately 500 μm deep in the dermal tissue, using × 20 objective and × 10 ocular lenses. The average BVD and LVD in the Paget disease group were calculated as the average number of vessels per field within an area of the epidermis containing Paget cells, as confirmed by cytokeratin 7 immunostaining. The average BVD and LVD in the control group were calculated as the average number of vessels per field within the entire nipple surface area. The average BVD and LVD in the dermatitis group were calculated as the average number of vessels per field within the entire dermis area of biopsy specimens.

The proportion of proliferating endothelial cells was calculated as the number of endothelial cells with Ki-67-stained nuclei divided by the total number of endothelial cells. The cell numbers were counted under high magnification (× 10 ocular and × 40 objective) in the same areas as those used for the vessel density measurements.

### Ratio of the blood vessel lumen area to nipple area

**Fig. 1:**
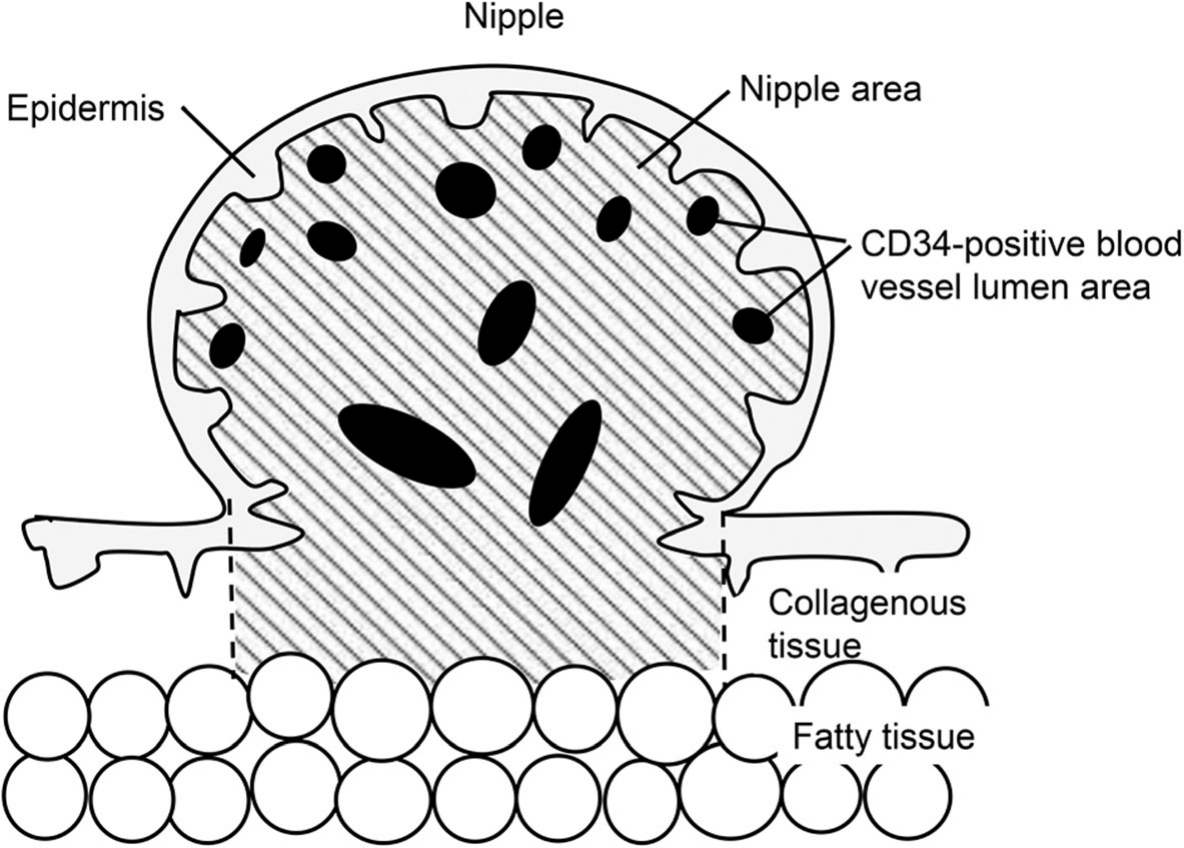
A schematic illustration of the “nipple area” defined in this study. The nipple area (shaded with oblique lines) was defined as follows: the lateral borders were defined as the lateral borders of the nipple, the upper border as the basement membrane of the epidermis, and the lower border as the border between the collagenous tissue of the dermis and subcutaneous fatty tissue. The black circles represent CD34-positive vessel structures. The ratio of the blood vessel lumen area to nipple area was defined as follows: (the summed area of all CD34-positive vessel structures / the nipple area) × 100

As a parameter related to blood flow in the nipple, the entire blood vessel lumen area relative to nipple area was measured in each case using an automated image analysis system (Visual Measure 32 software; Rise System, Sendai, Japan). The nipple area was defined according to the distance between the lateral borders of the nipple and the distance from the basement membrane of the epidermis to the border between the collagenous and subareolar fatty tissues (Fig. 1). The ratio of the blood vessel lumen area to nipple area was defined as follows: (the summed area of all CD34-positive vessel structures / the nipple area) × 100. The vessels included capillaries, arterioles, and venules.

### Immunohistochemical scoring of angiogenic factors

The immunohistochemical staining intensities of bFGF and VEGFA were scored as follows: 0, none; 1, weak; 2, moderate; 3, strong. The positively stained area was expressed as a percentage of the entire cancer area and scored as follows: 0, none; 1, 0–25%; 2, 25–50%; 3, > 50%. The immunoreactive score for each case was then calculated by multiplying the staining intensity score by the positively stained area score, yielding a possible value of 0–9.

### Statistical analysis

One-way analysis of variance was used to compare age among the groups. Median comparisons of the average BVD and LVD, proportion of proliferating endothelial cells, ratio of the blood vessel lumen area to nipple area, and immunohistochemical staining scores for bFGF and VEGFA among the Paget disease, dermatitis, and control groups were performed using the Kruskal–Wallis test, followed by the Mann–Whitney U test as a post-hoc test for comparisons between two groups. The correlations between these parameters were analyzed by Pearson’s correlation coefficient method. Statistical analyses were performed using commercially available software (IBM SPSS Statistics, version 24, IBM Corp., Armonk, NY, USA). Differences with *p* < 0.05 were considered statistically significant.

## Results

### Comparisons of blood and lymphatic vessel densities and proportion of proliferating endothelial cells among the Paget disease, dermatitis, and control groups

**Fig. 2:**
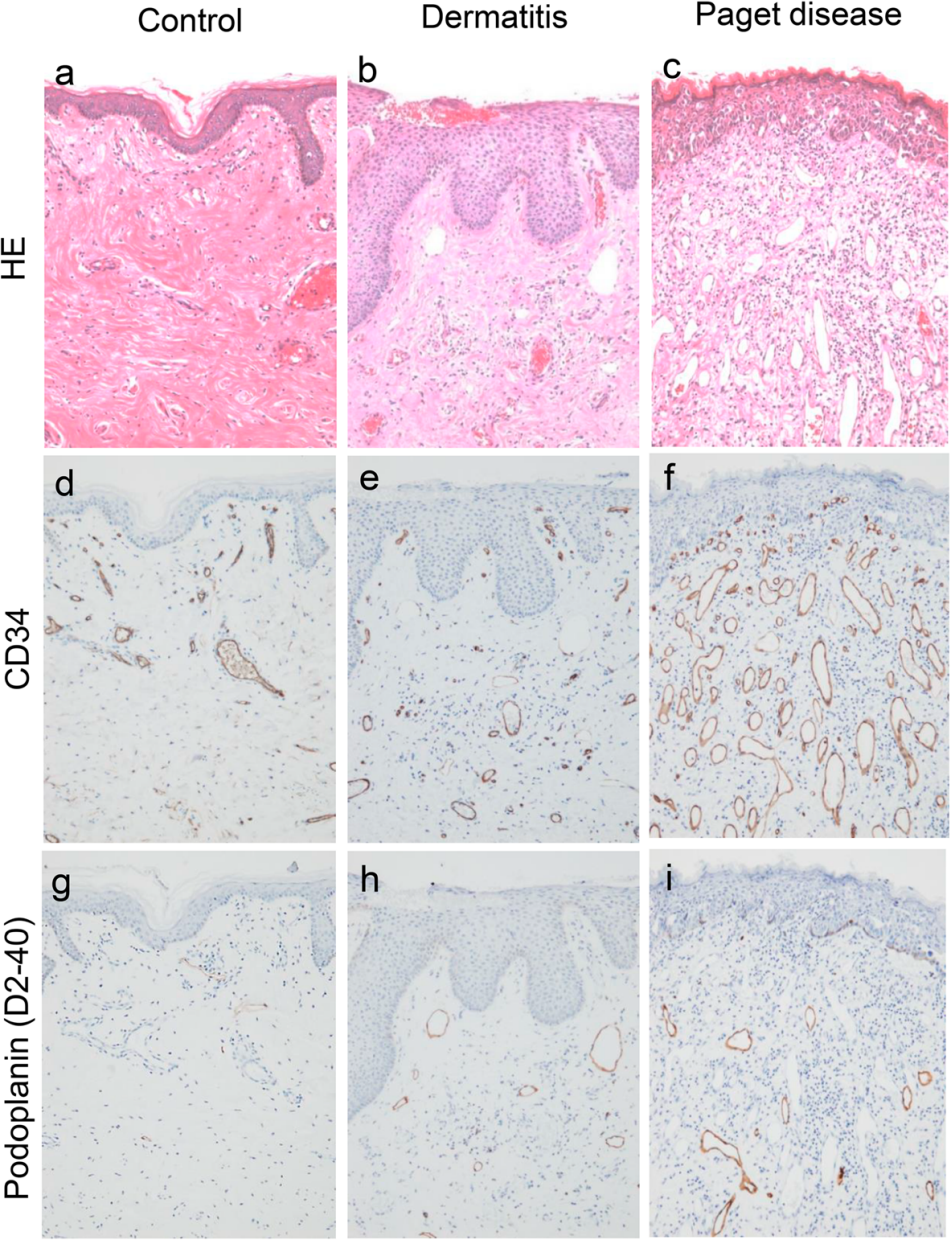
Hematoxylin and eosin (H&E) staining and immunohistochemical staining of CD34 and podoplanin (D2–40) in the nipple. **a**, **d**, **g** In a representative control case, only a few vessels are observed in the dermis (H&E staining (**a**), CD34 immunohistochemical staining (**d**), and podoplanin immunohistochemical staining (**g**)). **b**, **e**, **h** In a dermatitis case, a few vessels are observed with mild lymphocytic infiltration (H&E staining (**b**), CD34 immunohistochemical staining (**e**), and podoplanin immunohistochemical staining (**h**)). **c**, **f**, **i** In a mammary Paget disease case, Paget cells are observed in the epidermis, the number of vessels was markedly increased compared with the control and dermatitis cases, and lymphocytic infiltration is present in the dermis (H&E staining (**c**)). These vessels are predominantly CD34-positive (**f**), but several podoplanin-positive vessels are also seen (**i**). All magnification: × 100

In the superficial area of the dermis, a few vessels were apparent in the control group (Fig. 2a, d, and g). Slightly more vessels as well as epidermal thickening and mild lymphocytic infiltration were observed in the dermis of the dermatitis group compared with the control group (Fig. 2b, e, and h). In the Paget disease group, the vessels were increased in size and number, particularly in the superficial area of the dermis, compared with the control and dermatitis groups (Fig. 2c). None of the vessels in the superficial area of the dermis had muscular media or elastic lamella, but all were covered completely by αSMA-positive cells (pericytes). The structure of these vessels did not show any abnormalities. In the superficial nipple area, the majority of the vessels were CD34-positive blood vessels, whereas podoplanin (D2–40)-positive lymphatic vessels comprised a lower proportion (Fig. 2f and i). In the dermis beneath the proliferating Paget cells, not only increased blood and lymphatic vessel numbers but also various degrees of lymphocytic infiltration were observed. In the deep nipple tissue, the blood vessels comprised mainly arterioles and venules with muscular media; thus, the number of blood vessels was almost equal between the Paget disease and control groups in this tissue.

**Fig. 3:**
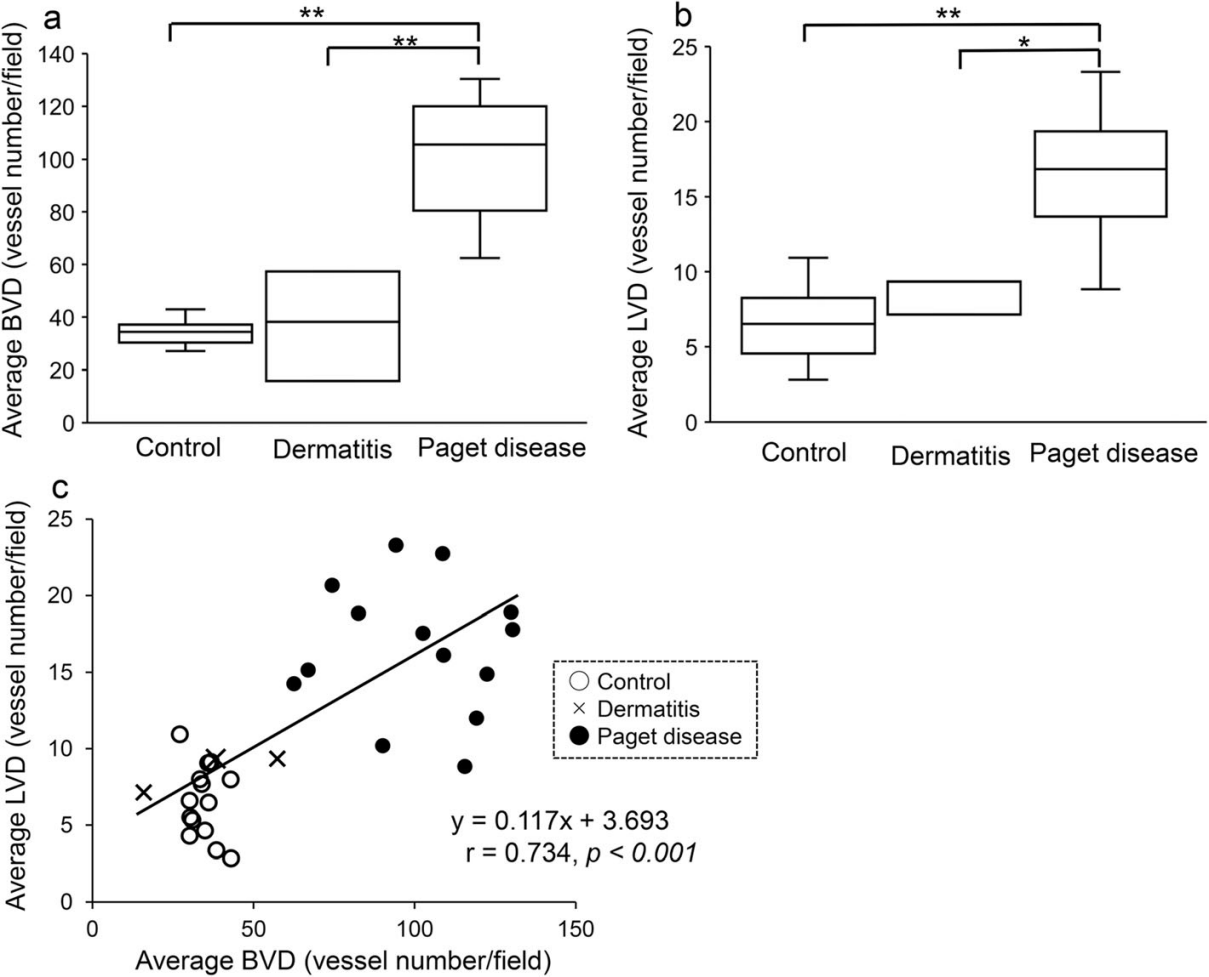
Average blood vessel density (BVD) and lymphatic vessel density (LVD) in the control, dermatitis, and Paget disease groups. **a** The average BVD was significantly greater in the Paget disease group than in the control (**p < 0.001) and dermatitis (**p < 0.001) groups. **b** The average LVD was significantly greater in the Paget disease group than in the control (**p < 0.001) and dermatitis (*p < 0.05) groups. However, no difference in the average BVD (*p* = 0.676) or LVD (*p* = 0.121) was found between the control and dermatitis groups. **c** The average BVD was strongly correlated with the average LVD (*r* = 0.734, *p* < 0.001)

The Kruskal–Wallis and post-hoc Mann–Whitney U tests showed that both the average BVD were significantly greater in the Paget disease group than in the control (105.6 ± 21.8 vs. 34.4 ± 4.5, *p* < 0.001) and dermatitis groups (105.6 ± 21.8 vs. 38.2 ± 16.9, *p* = 0.003), respectively (Fig. 3a). Similarly, the average LVD were significantly greater in the Paget disease group than in the control (16.8 ± 4.2 vs. 6.5 ± 2.3, *p* < 0.001) and dermatitis groups (16.8 ± 4.2 vs. 9.3 ± 1.0, *p* = 0.012), respectively (Fig. 3b). The average BVD was strongly correlated with the average LVD (*r* = 0.734, *p* < 0.001) (Fig. 3c).

The proportion of proliferating endothelial cells was significantly higher in the Paget disease group (2.683 ± 2.400) than in the control group (0.150 ± 0.132, *p* = 0.001) (Supplemental Fig. 1), but the increase in the dermatitis group (1.011 ± 1.194) compared with the other groups was not significant. The proportion of proliferating endothelial cells was significantly correlated with the average BVD (*r* = 0.680, *p* < 0.001).

### Comparison of the ratio of the blood vessel lumen area to nipple area and proportion of proliferating endothelial cells between the Paget disease and control groups

**Fig. 4:**
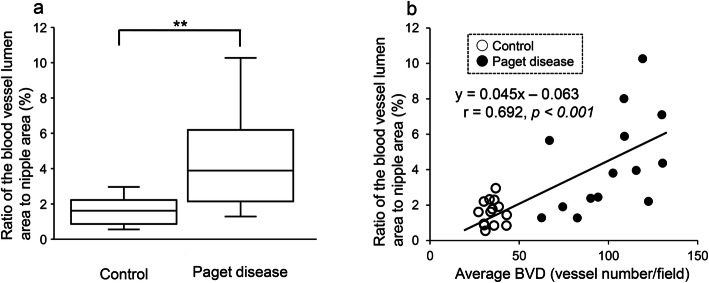
(**a**) The ratio of the blood vessel lumen area to nipple area was significantly greater in the Paget disease group than in the control group (**p < 0.01) and (**b**) was significantly correlated with the average BVD (*r* = 0.692, *p* < 0.001)

The ratio of the blood vessel lumen area to nipple area was significantly greater in the Paget disease group than in the control group (4.3 ± 2.7 vs. 1.6 ± 0.7, *p* = 0.003) (Fig. 4a). In addition, this ratio was correlated with the average BVD (*r* = 0.692, *p* < 0.001) (Fig. 4b), average LVD (*r* = 0.480, *p* = 0.010) and proportion of proliferating endothelial cells (*r* = 0.507, *p* = 0.006).

### Immunohistochemical analysis of angiogenic factors

**Fig. 5:**
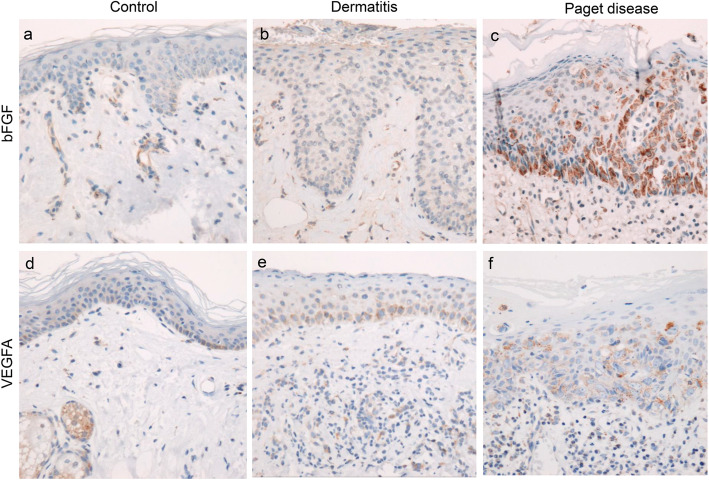
Immunohistochemical expression of basic fibroblast growth factor (bFGF) (**a**-**c**, × 200) and vascular endothelial growth factor A (VEGFA) (**d**-**f**, × 200). In control (**a**) and dermatitis (**b**) tissues, bFGF expression is absent in non-neoplastic epithelium and weak in the endothelium of the dermis. In Paget disease tissues (**c**), immunohistochemical bFGF expression is diffuse in the cytoplasm of Paget cells. VEGFA cytoplasmic expression is weak to moderate in non-neoplastic epithelium such as epidermis and sebaceous glands in the control (**d**) and dermatitis (**e**) groups, and weak to strong in Paget cells of the Paget disease group (**f**)

**Fig. 6:**
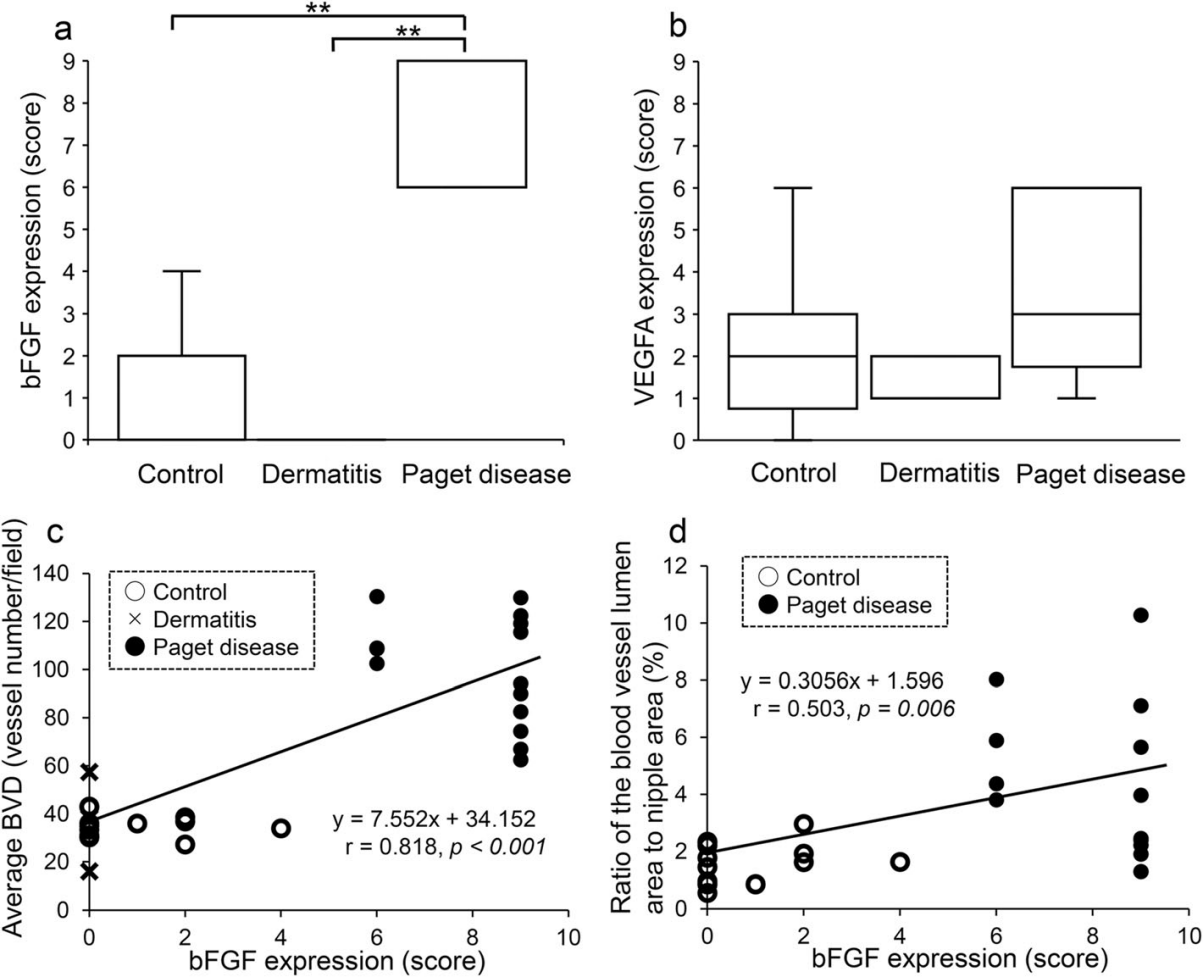
The expression of basic fibroblast growth factor (bFGF) and vascular endothelial growth factor A (VEGFA) in the control, dermatitis, and Paget disease groups. **a** bFGF expression was significantly higher in the Paget disease group than in the control and dermatitis groups (**p < 0.001, respectively). **b** VEGFA expression showed no significant difference among the three groups. The correlations of bFGF expression with **c** the average blood vessel density (BVD) and **d** the ratio of the blood vessel lumen area to nipple area. bFGF expression was strongly correlated with the average BVD (*r* = 0.818, *p* < 0.001) and the ratio of the blood vessel lumen area to nipple area (*r* = 0.503, *p* = 0.006)

In the control and dermatitis groups, immunohistochemical expression of bFGF was almost absent in non-neoplastic epithelium and was weak in endothelial cells (Fig. 5a and b). In contrast, in the Paget disease group, bFGF was diffusely expressed in the cytoplasm of Paget cells (Fig. 5c). Cytoplasmic expression of VEGFA was weak to moderate in non-neoplastic epithelial cells, such as mammary and sebaceous glands (Fig. 5d and e), and weak to strong in Paget cells (Fig. 5f). The bFGF expression score was significantly higher in the Paget disease group (8.1 ± 1.4) than in the control (0.8 ± 1.2, *p* < 0.001) and dermatitis (0.0 ± 0.0, *p* = 0.003) groups (Fig. 6a). The VEGFA expression score tended to be higher in the Paget disease group (3.4 ± 1.9) than in the control group (2.1 ± 1.5, *p* = 0.104), but the difference was not statistically significant (Fig. 6b). In addition, the bFGF expression score was significantly correlated with the average BVD (*r* = 0.818, *p* < 0.001) (Fig. 6c), average LVD (*r* = 0.763, *p* < 0.001), proportion of proliferating endothelial cells (*r* = 0.498, *p* = 0.004) and ratio of the blood vessel lumen area to nipple area (*r* = 0.503, *p* = 0.006) (Fig. 6d). In contrast, the VEGFA expression score was not correlated with the average BVD (*r* = 0.203, *p* = 0.273), average LVD (*r* = 0.253, *p* = 0.170), proportion of proliferating endothelial cells (*r* = 0.166, *p* = 0.372), or ratio of the blood vessel lumen area to nipple area (*r* = 0.114, *p* = 0.565), but was positively correlated with the bFGF score (*r* = 0.410, *p* = 0.006).

## Discussion

In mammary Paget disease, the number of blood vessels was increased significantly compared with the control and dermatitis groups. In addition, the ratio of the blood vessel lumen area to nipple area, a parameter used to estimate blood flow volume, was increased markedly in the Paget disease group. bFGF expression was robust in Paget cells and was strongly correlated with the average BVD and ratio of the blood vessel lumen area to nipple area, whereas the relationship between VEGFA expression and vascular density was not statistically significant.

In this study, a dramatic increase in the number of CD34-positive blood vessels, with a minor proportion of podoplanin (D2–40)-positive lymphatic vessels, was observed in the Paget disease group. Additionally, we identified significant proliferation of endothelial cells in the Paget disease group. Increased blood flow in Paget disease in MRI and Doppler sonography imaging studies has been well documented [4–6]. However, three studies of angiogenesis in Paget disease did not examine tumor tissue vascularity and only demonstrated the expression of some angiogenic factors [2, 8, 9]; very few histological studies of the vascularity in mammary Paget disease have been conducted [6, 7]. Ellis et al. reported that microvessel density, according to immunohistochemical staining of von Willebrand factor, was significantly greater in mammary Paget disease with invasive ductal carcinoma or ductal carcinoma in situ than in mammary Paget disease alone [7]. However, those authors did not compare microvascular density between Paget disease and normal/inflammatory nipple lesions. Our study included a limited number of cases, especially of nipple dermatitis. However, comparisons among normal, dermatitis, and mammary Paget disease cases are important to clarify the difference between nipple dermatitis and Paget disease. We found a significantly greater BVD in the Paget disease group than in the control and dermatitis groups. In contrast, we found no significant difference in BVD between pure Paget disease and Paget disease with invasive ductal carcinoma or ductal carcinoma in situ (data not shown).

One of the reasons for the discrepancy between our results and those of Ellis et al. may be the method used to assess vessel density. Ellis et al. included only microvessels in their measurements, excluding large vessels with lumen diameters greater than approximately 7 erythrocytes [7]. On the other hand, we counted all CD34-positive blood vessels in the dermal tissue to a depth of 500 μm, because our purpose was to analyze the histological findings related to blood flow volume in the nipple. In addition, we confirmed that not only the number of blood vessels but also the ratio of the blood vessel lumen area to nipple area was significantly increased in Paget disease.

As an early event in the neoplastic process, the “initiation switch” of angiogenesis [10] is represented by the response to angiogenic factors released by the cells adjacent to a carcinoma [11]. Increased blood flow in the nipple may lead to early detection of mammary Paget disease. Despite our limited sample size, the findings from this study support the enhanced blood flow observed previously by MRI [4, 5] and Doppler sonography [6] in Paget disease. These clinical imaging findings are now considered useful parameters for diagnosing mammary Paget disease.

Paget cells strongly expressed bFGF in the cytoplasm in all cases of our study. This growth factor is a potent inducer and positive regulator of angiogenesis via recruitment of inflammatory cells [12]. Xu et al. reported that the immunohistochemical staining score and mRNA expression level of bFGF were greater in extramammary Paget disease than in normal tissues [2], but the relationship between bFGF expression and vascularity was not assessed. We detected bFGF expression in Paget cells as well as positive correlations between the bFGF expression level and both the average BVD and ratio of the blood vessel lumen area to nipple area. Incidentally, bFGF is not only an angiogenic factor but also a lymphangiogenic factor [13], and lymphangiogenesis in extramammary Paget disease has been reported [14]. Indeed, our results demonstrated a significant increase in the average LVD in Paget disease and its positive correlation with bFGF expression; however, podoplanin (D2–40)-positive lymphatic vessels comprised a minor proportion of the vessels compared with the CD34-positive blood vessels. Thus, our results support the concept that bFGF is a predominant inducer of angiogenesis in mammary Paget disease.

In contrast, the relationship between VEGFA expression and Paget disease is controversial. Ellis et al. reported that VEGFA is not expressed in Paget disease of the breast and vulva [9], but other studies showed VEGF expression in Paget cells [2, 8]. In our results, because varying degrees of VEGF expression were observed in both Paget and non-Paget disease cells, the evaluation of VEGFA expression was difficult. However, bFGF may require activation of the VEGF/FGF receptor system to promote angiogenesis [2, 12], and a significant correlation between the mRNA expression levels of bFGF and VEGF has been reported in extramammary Paget disease tissues [2]. In the present study, the VEGFA expression score tended to be higher in the Paget disease group than in the control group, although the difference was not statistically significant. Although immunohistochemical expression of the VEGF receptor could not be confirmed in the present study, evaluation of VEGF receptor expression in future studies may be useful for further understanding vascular proliferation in mammary Paget disease. Furthermore, a significant correlation between bFGF and VEGFA expression was detected immunohistochemically. Therefore, angiogenesis in mammary Paget disease was promoted by bFGF predominantly, but VEGFA may cooperate with bFGF to achieve this effect.

## Conclusions

The number of dermal blood vessels in mammary Paget disease was dramatically increased compared with the control and dermatitis cases. Additionally, the ratio of the blood vessel lumen area to nipple area was greater in Paget disease than in the controls. These parameters were significantly correlated with bFGF expression in Paget cells, and tended to be correlated with VEGFA expression. Therefore, we histopathologically confirmed the increased nipple blood flow in Paget disease detected by clinical image analyses such as MRI and Doppler sonography, which have been proposed as well-substantiated markers distinguishing mammary Paget disease from normal and dermatitis cases and for detecting Paget disease at an early stage.

## Supplementary information

**Additional file 1: Supplemental Figure 1.** Immunohistochemical double staining of CD34 (red) and Ki-67 (brown). Red cells are CD34-positive endothelial cells. The endothelial cells with brown nuclei are considered to be proliferating (arrow). (A) In a control case, only one brown nucleus in an endothelial cell is observed. (B) In a case of mammary Paget disease, many proliferating endothelial cells are observed. Epidermal basal layer cells are used as a positive internal control. All magnifications: × 400.

## Data Availability

The datasets generated and /or analysed during the current study are not publicly available due the institutional review board restricts the use of the datasets to the current study only.

## Abbreviations

bFGF: Basic fibroblast growth factor
BVD: Blood vessel density
LVD: Lymphatic vessel density
MRI: Magnetic resonance imaging
VEGFA: Vascular endothelial growth factor A
