# Supplementary figures and images for: Angiogenesis in mammary Paget disease: histopathological analyses of blood vessel density and angiogenic factors
Source: Diagn Pathol. 2020 Jun 11;15:75. doi: 10.1186/s13000-020-00988-y (PMC7288480; doi:10.1186/s13000-020-00988-y)

Control

Paget disease

A

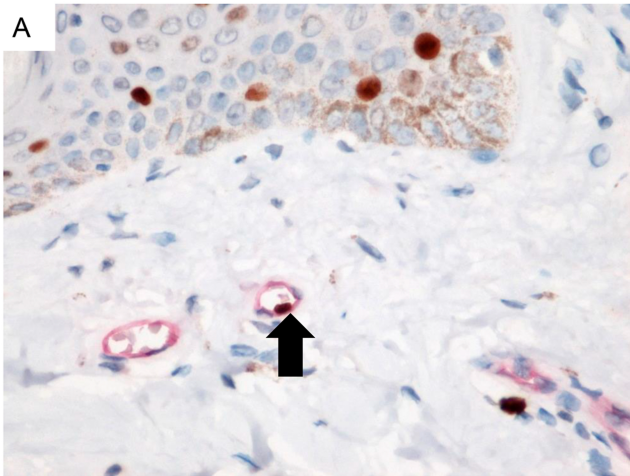

B

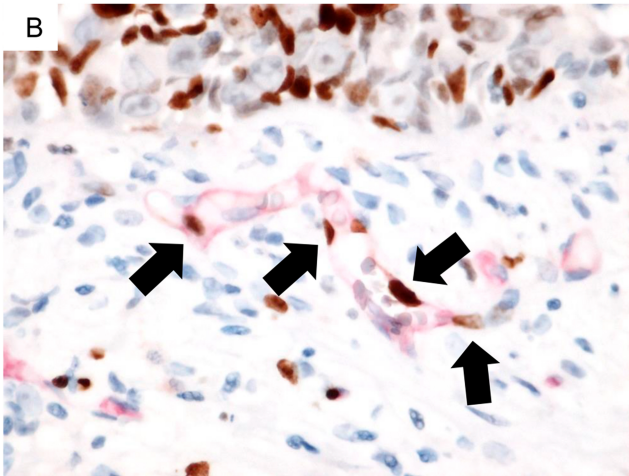

Supplement: Supplementary file 1 — Additional file 1: Supplemental Figure 1. Immunohistochemical double staining of CD34 (red) and Ki-67 (brown). Red cells are CD34-positive endothelial cells. The endothelial cells with brown nuclei are considered to be proliferating (arrow). (A) In a control case, only one brown nucleus in an endothelial cell is observed. (B) In a case of mammary Paget disease, many proliferating endothelial cells are observed. Epidermal basal layer cells are used as a positive internal control. All magnifications: × 400. [file 13000_2020_988_MOESM1_ESM.pdf]
